# Birth weight-for-gestational age is associated with DNA methylation at birth and in childhood

**DOI:** 10.1186/s13148-016-0285-3

**Published:** 2016-11-16

**Authors:** Golareh Agha, Hanine Hajj, Sheryl L. Rifas-Shiman, Allan C. Just, Marie-France Hivert, Heather H. Burris, Xihong Lin, Augusto A. Litonjua, Emily Oken, Dawn L. DeMeo, Matthew W. Gillman, Andrea A. Baccarelli

**Affiliations:** 1 Department of Environmental Health Sciences, Mailman School of Public Health, Columbia University, 722 West 168th Street, New York, NY 10032 USA; 2 Division of Newborn Medicine, Boston Children’s Hospital, Boston, MA USA; 3 Obesity Prevention Program, Department of Population Medicine, Harvard Medical School and Harvard Pilgrim Health Care Institute, Boston, MA USA; 4 Department of Preventive Medicine, Icahn School of Medicine at Mount Sinai, New York, NY USA; 5 Diabetes Unit, Massachusetts General Hospital, Boston, MA USA; 6 Department of Neonatology, Beth Israel Deaconess Medical Center, Department of Pediatrics, Harvard Medical School, Boston, MA USA; 7 Department of Environmental Health, Harvard T.H. Chan School of Public Health, Boston, MA USA; 8 Department of Biostatistics, Harvard T.H. Chan School of Public Health, Boston, MA USA; 9 Channing Laboratory, Brigham and Women’s Hospital, Boston, MA USA; 10 Channing Division of Network Medicine, Brigham and Women’s Hospital, Harvard Medical School, Boston, MA USA; 11 Department of Nutrition, Harvard T.H. Chan School of Public Health, Boston, MA USA

**Keywords:** Epigenetics, DNA methylation, Birth weight

## Abstract

**Background:**

Both higher and lower fetal growth are associated with cardio-metabolic health later in life, suggesting that prenatal developmental programming determines long-term cardiovascular disease risk. Epigenetic mechanisms, which orchestrate fetal growth and development, may offer insight on the early programming of health and disease. We investigated whether birth weight-for-gestational is associated with DNA methylation at birth and mid-childhood, measured via the Infinium 450K array.

**Methods/results:**

Participants were from Project Viva, a pre-birth cohort of pregnant women and their children in Eastern Massachusetts. After exclusion of participants with maternal type 1 or 2 diabetes and gestational age <34 weeks, we used DNA methylation assays from 476 venous umbilical cord blood samples and a subset of 235 who additionally had peripheral blood samples available in mid-childhood (age 7–10 years). Among 392,918 CpG sites analyzed, birth weight-for-gestational age *z*-score was associated with cord blood DNA methylation at 34 CpGs (false discovery rate *P* < 0.05), after adjusting for maternal age, race/ethnicity, education, smoking, parity, delivery mode, pre-pregnancy BMI, gestational diabetes status, child sex, and estimated cord blood cell proportions based on a cord blood reference panel. Two of these CpGs were previously reported in epigenome-wide analyses of birth weight, and several other CpGs map to genes relevant to fetal growth and development. Namely, higher birth weight-for-gestational age was associated with higher methylation at four CpGs at the *PBX1* locus (e.g., β (95% CI) for lead signal at cg06750897 = 1.9 (1.2, 2.6)), which encodes a transcription factor that regulates embryonic development. Birth weight-for-gestational age was also associated with mid-childhood blood DNA methylation at four of the 34 CpGs identified in cord blood analyses, including sites at the *PBX1* locus described.

**Conclusions:**

We identified CpG sites where birth weight-for-gestational age was associated with DNA methylation at birth, and for a subset of these sites, birth weight-for-gestational age was also associated with DNA methylation at mid-childhood.

**Electronic supplementary material:**

The online version of this article (doi:10.1186/s13148-016-0285-3) contains supplementary material, which is available to authorized users.

## Background

Fetal growth predicts both short- and long-term health, including cardio-metabolic health. Low birth weight has been associated with higher blood pressure, insulin resistance, type 2 diabetes, coronary heart disease events, and cardiovascular mortality later in life [1–4], and there is strong evidence that these associations are particularly due to impaired fetal growth [5, 6]. Conversely, studies of high birth weight have shown an association with higher subsequent risk of obesity [7, 8]. The link between fetal growth and later life cardio-metabolic events remains poorly understood. Identification of molecular markers that are measured early and persist over time may provide insight into developmental origins of chronic cardio-metabolic diseases.

Epigenetic mechanisms such as DNA methylation play a central role in fetal growth and development [9]. Furthermore, an adverse in utero environment can influence establishment of epigenetic patterning and affect fetal development [10, 11]. Several studies have shown associations between DNA methylation patterns and exposures during the in utero period, such as maternal famine, smoking, and diet [12–16]. Therefore, DNA methylation patterns associated with an indicator of fetal growth, such as birth weight adjusted for gestational age, may serve as epigenetic markers of an adverse fetal environment and help elucidate the early programming of associated cardio-metabolic risk.

A handful of initial studies in humans have revealed associations between birth weight and DNA methylation [17–21]. These past studies have either mainly focused on targeted genomic regions, have been relatively small in size, have not adequately accounted for gestational age, or have looked at DNA methylation at only one time point. More recently, Engel et al. performed a comprehensive epigenome-wide scale analyses in a large homogeneous Norwegian study population from the MoBa cohort, and reported associations of birth weight (independent of gestational age) with cord blood DNA methylation at 19 CpG sites [22]. However, their study also examined DNA methylation at only one time point.

If DNA methylation is a stable marker of fetal programming, then the association of fetal growth with DNA methylation patterns might be expected to persist over time. In the longitudinal ALSPAC cohort in South West England, birth weight (independent of gestational age) was associated with cord blood DNA methylation in 23 CpG sites [23]. The investigators further used longitudinal analyses and observed that methylation levels changed at the majority of these sites, concluding that birth weight-associated differential methylation does not persist with time. However, other data provide evidence that suggests persistence of DNA methylation effects in response to in utero environmental conditions. In samples collected 60 years after the Dutch Hunger Winter, there were DNA methylation differences between individuals who were prenatally exposed to in utero famine in comparison to their unexposed same-sex siblings [12]. These DNA methylation changes were observed in biological pathways related to growth and metabolism and in genes associated with birth weight [24]. Currently, whether associations of fetal growth with DNA methylation persist over time remains an open question.

Finally, it is important to note that prior epigenetic association studies in cord blood have made statistical adjustments for cell type proportions using an adult peripheral blood methylation reference panel [22, 23]. However, this may not be appropriate for epigenetic studies of cord blood [25]. According to recent evidence, the distribution [26] and methylation profiles [27] of cord blood cell types are distinct and differ from blood at later ages. This is particularly the case for nucleated red blood cells (nRBCs), which are commonly present only in cord blood, and also appear in buffy coat isolated from cord blood. Thus, it is important to account for cord blood cell type proportions in epigenome-wide analyses by using an appropriate reference panel.

We conducted an epigenome-wide DNA methylation analysis to examine the extent to which birth weight-for-gestational age (BW/GA) is associated with DNA methylation at birth, using cord blood DNA methylation profiles in 476 individuals from the Project Viva cohort. We adjusted for cord blood cell type proportions using the cord blood methylation reference panel recently made available by Bakulski et al. [27], which was also recently validated against directly measured cell type composition in cord blood [28]. For CpG sites where BW/GA was associated with cord blood DNA methylation, we further investigated the extent to which BW/GA was associated with peripheral blood DNA methylation at mid-childhood.

## Methods

### Study population

Study participants were from Project Viva, a prospective observational cohort study in Eastern Massachusetts that recruited pregnant women from 1999 to 2002 [29]. Research personnel recruited women at their first prenatal visit at one of eight obstetric offices of Atrius Harvard Vanguard Medical Associates, a multi-specialty group practice. Eligibility requirements were the ability to answer questions in English, at <22 weeks of gestation at study entry, and a singleton pregnancy. All women provided written informed consent, and institutional review boards of participating institutions approved the study [29]. Of 2218 live births, we collected 1018 venous umbilical cord blood samples at the time of delivery. Of these, cord blood DNA methylation assays were completed in 2014 for 507 Viva infants with genetic consent, of whom 22 were excluded due to low quality or irreconcilable sample swaps. Among remaining participants (*n* = 485), we excluded infants if mothers had type 1 diabetes (*n* = 1), type 2 diabetes (*n* = 1), missing covariate info (*n* = 1 missing pre-pregnancy body mass index, BMI), or if the infant’s gestational age at delivery was <34 weeks (*n* = 6). The final analytic sample for cord blood analyses was 476. Of these 476 participants, 235 also had DNA methylation samples (assayed concurrently with cord blood samples) from mid-childhood (mean 7.9 years, range 6.7–10.5 years) peripheral white blood cells.

### Ascertainment of birth data and measurement of birth weight-for-gestational age

We obtained infant birth weight in grams and date of delivery from hospital medical record. We calculated length of gestation in days by subtracting the date of the last menstrual period (LMP) from the date of delivery. If gestational age according to the second-trimester ultrasound differed from that according to the LMP by >10 days, we used ultrasound dating to determine gestational age. We determined sex-specific BW/GA *z*-scores from a US national reference [30].

### Covariates

Research personnel used interviews, mailed questionnaires, and clinical records to obtain information on maternal characteristics, including race/ethnicity (non-Hispanic white, black, Hispanic, Asian, or other), educational status (less than high school, high school diploma, some college, BA or BS, or graduate degree), smoking status (never, former, smoked any time during pregnancy), maternal age (reported at enrollment), maternal pre-pregnancy BMI (based on self-report at enrollment of height and pre-pregnancy weight), parity, mode of delivery (cesarean or vaginal delivery), gestational diabetes status (obtained from prenatal clinical records on maternal glucose tolerance testing; categorized as normal, isolated hyperglycemia, gestational impaired glucose tolerance, or gestational diabetes). For the current analyses, we collapsed “Asian” and “other” to include race/ethnicity as a 4-category variable, and we dichotomized educational status as college graduate vs. not a college graduate, and parity as 0 (nulliparous) vs. 1 or more (multiparous).

### Measurement, filtering, and processing of DNA methylation data

Trained medical personnel obtained venous umbilical cord blood samples immediately after delivery, which they promptly stored in a dedicated refrigerator (4 °C) and transported for processing within 24 h, and trained laboratory staff processed the samples on the same day. Whole blood samples were centrifuged to separate the buffy coat from plasma and red blood cells (RBCs), and the buffy coat was transferred into an RBC lysis solution to facilitate further lysis of RBCs. The solution was then centrifuged to obtain white blood cell (WBC) pellet and remove the lysis solution containing RBCs. A similar protocol was followed for peripheral blood samples at mid-childhood. DNA was extracted using the Qiagen Puregene Kit (Valencia, CA). Aliquots were then stored at −80 °C until analysis. DNA was sodium bisulfite converted using the EZ DNA Methylation-Gold Kit (Zymo Research, Irvine, CA). We used a two-stage algorithm to randomly allocate samples to plates and chips in a manner ensuring balance by sex, and analyzed the samples using the Infinium Human Methylation450 BeadChip array (Illumina, San Diego, CA). For each CpG site, methylation = *M*/(*M* + *U* + *ε*), where *M* and *U* refer to the average fluorescence intensity from the probe (i.e., oligonucleotide that hybridizes to the target CpG) corresponding to the methylated and unmethylated target CpG, respectively, and *ε* = 100 to protect against division by zero. Therefore, methylation at each CpG can range from 0 to 1, with 0 indicating no methylation and 1 indicating 100% methylation.

We performed data import and pre-processing using R and Bioconductor package methylumi [31].

In addition to dropping low-quality samples, we excluded probes that had a detection *p* value >0.05 for more than 1% of the samples (i.e., a signal was not detected from that probe). We additionally removed non-CpG probes, sex chromosome probes, and polymorphic probes (defined as SNP-overlapping probes, probes with a SNP at the target CpGs, or probes with a SNP at the base next to the target CpG) with minor-allele frequency (MAF) ≥5%; based on UCSC common SNPs track for dbSNP build 137. We further removed any remaining probes that are considered cross-hybridizing [32]. We applied this stringent CpG-filtering because polymorphic and cross-hybridizing probes can interfere with accurate detection of methylation levels [32]. The final number of probes included in the analyses was 392,918. We then performed background adjustment via the normal-exponential out-of-band (“noob”) background correction method with dye-bias equalization [33], and further normalized using the Beta-Mixture Quantile dilation (BMIQ) approach [34]. We visually examined strip plots of control probes for bisulfite conversion and specificity, and examined density plots for the β-values across samples at each normalization step. We applied the ComBat method to adjust the methylation data for sample plate, to reduce potential for bias due to batch effects [35].

### Statistical analyses

For epigenome-wide analyses, we logit-transformed the methylation values to obtain methylation data on the M-value scale; this better satisfies assumptions of linear regression and is more statistically valid for differential methylation analyses [36]. We used robust linear regression models to conduct CpG-by-CpG analyses, with logit-transformed M-values as the dependent variable and BW/GA *z*-score as the continuous independent variable. Analyses were adjusted for potential confounders of the BW/GA–DNA methylation association, including maternal age (continuous), race, education, smoking status, parity, mode of delivery, pre-pregnancy BMI (continuous), gestational diabetes status, and child sex. To adjust for blood cell type proportions, we used the statistical deconvolution method of Houseman et al. [37]. For our cord blood analyses, we used a reference panel of nucleated cells isolated from cord blood [27]. We corrected for multiple testing by controlling the false discovery rate at 5%, thus we considered an associations with FDR *q* value <0.05 as significant.

We then conducted analyses of BW/GA *z*-score and mid-childhood peripheral blood DNA Methylation, limited to the CpGs that were significant in the cord blood analyses. Mid-childhood peripheral blood analyses were adjusted for all covariates that were included in the cord blood analyses, and additionally adjusted for childhood age at the time of blood sampling, which ranged from 6.7 to 10.5 years. We used an adult leukocyte reference panel [38] for cell type adjustment in our mid-childhood analyses on peripheral blood*.* Estimates of cell type proportion were included as variables directly in the regression models. While the regression analyses were conducted with DNA methylation on the M-Value scale, effect estimates in the result are reported on the original scale, for easier interpretation. Thus, effect estimates represent difference in % methylation for a 1-unit increase in BW/GA *z*-score.

## Results

Among the 476 mother-infant pairs included in these analyses, mean (SD) maternal age was 32.1 (5.4) years at enrollment in early pregnancy. Approximately 71% of women were non-Hispanic white, 11.8% African-American, 7.8% Hispanic, and 9.5% as other race/ethnicity (including Asian and those identifying as more than one race). Additionally, 66% of women were college graduates, 11% reported smoking during pregnancy, and 36% were overweight or obese before pregnancy. Among infants, mean (SD) birth weight was 3561 (506) g, mean (SD) BW/GA *z*-score was 0.27 (0.96); 5% were small-for-gestational age (SGA; defined as BW/GA <10th percentile) and 15% were large-for-gestational age (LGA; defined as BW/GA ≥90th percentile); 48% of infants were female.

Mothers who were overweight or obese, or were multiparous, tended to give birth to infants with higher BW/GA (Table 1).

**Table 1:** Associations of maternal and infant characteristics with birth-weight-for-gestational-age (BW/GA) *z*-score in project Viva infants (*n* = 476)

	*N* (%)	Unadjusted effect estimate (95% CI)	Adjusted^a^ effect estimate (95% CI)
Maternal age at enrollment, years			
<25	46 (9.7)	−0.57 (−0.88,−0.27)	−0.22 (−0.56, 0.13)
25–<30	99 (20.8)	−0.10 (−0.33, 0.13)	−0.02 (−0.25, 0.21)
30–<35	192 (40.3)	0.0 (ref)	0.0 (ref)
35–<40	108 (22.7)	−0.02 (−0.24, 0.21)	−0.09 (−0.31, 0.14)
≥40	31 (6.5)	−0.06 (−0.42, 0.30)	−0.14 (−0.49, 0.22)
Maternal race/ethnicity			
White	338 (71.0)	0.0 (ref)	0.0 (ref)
Black	56 (11.8)	−0.29 (−0.56,−0.02)	−0.26 (−0.55, 0.02)
Hispanic	37 (7.8)	−0.16 (−0.48, 0.17)	−0.15 (−0.48, 0.18)
Other	45 (9.5)	−0.31 (−0.61,−0.02)	−0.15 (−0.45, 0.15)
Education			
Less than college graduate	161 (33.8)	−0.16 (−0.34, 0.02)	−0.16 (−0.37, 0.05)
≥College graduate	315 (66.2)	0.0 (ref)	0.0 (ref)
Maternal pre-pregnancy BMI category			
<18.5	18 (3.8)	−0.40 (−0.85, 0.06)	−0.33 (−0.78, 0.12)
18.5–<25	285 (59.9)	0.0 (ref)	0.0 (ref)
25–<30	105 (22.1)	0.26 (0.05, 0.48)	0.28 (0.06, 0.49)
≥30	68 (14.3)	0.27 (0.01, 0.52)	0.28 (0.02, 0.54)
Smoking status			
Never	324 (68.1)	0.0 (ref)	0.0 (ref)
Former	100 (21.0)	0.03 (−0.18, 0.25)	−0.07 (−0.28, 0.14)
During pregnancy	52 (10.9)	−0.20 (−0.48, 0.08)	−0.12 (−0.41, 0.17)
Maternal glucose tolerance			
Normal	391 (82.1)	0.0 (ref)	0.0 (ref)
Isolated hyperglycemia	45 (9.5)	0.28 (−0.01, 0.58)	0.24 (−0.06, 0.53)
Impaired glucose tolerance or gestational diabetes	40 (8.4)	0.26 (−0.05, 0.57)	0.19 (−0.12, 0.50)
Parity			
≥1	254 (53.4)	0.42 (0.25, 0.59)	0.41 (0.23, 0.59)
0	222 (46.6)	0.0 (ref)	0.0 (ref)
Mode of delivery			
Vaginal	397 (83.4)	0.00 (−0.24, 0.23)	0.04 (−0.18, 0.27)
Cesarean section	79 (16.6)	0.0 (ref)	0.0 (ref)
Child sex			
Male	248 (52.1)	−0.07 (−0.25, 0.10)	−0.07 (−0.24, 0.10)
Female	228 (47.9)	0.0 (ref)	0.0 (ref)

^a^Estimates of effect were simultaneously adjusted for all other characteristics in the table

**Fig. 1:**
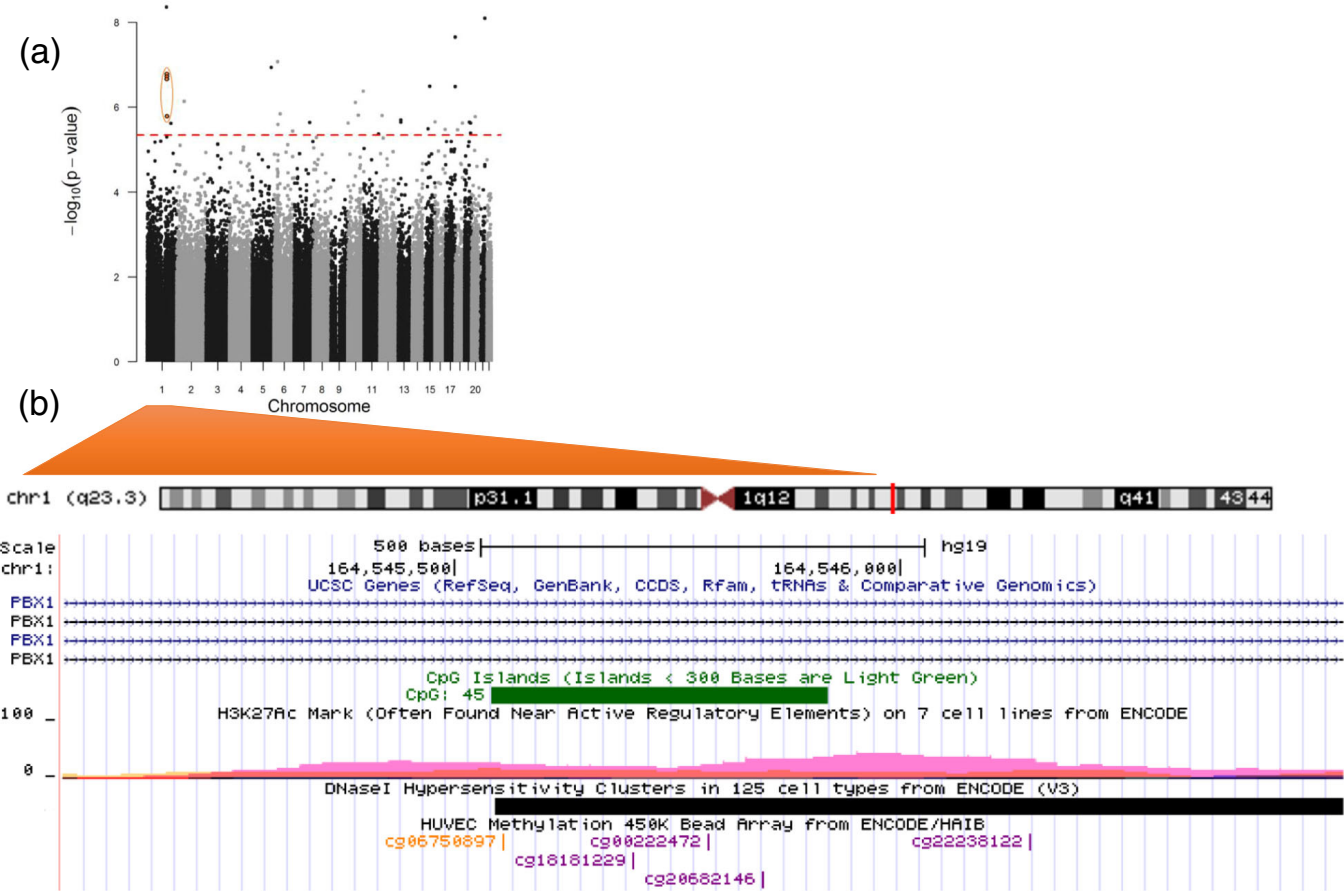
**a** Manhattan plot for the association of birth weight-for-gestational age (BW/GA) with epigenome-wide cord blood DNA methylation (*circled orange dots* indicate the *PBX1* CpGs: cg18181229, cg06750897, cg00222472, cg20682146). **b** Magnified depiction of the *PBX1* gene region within chromosome 1, with annotated genomic tracks: CpG-island location (*green box*), H3K27Ac histone mark enrichment levels (*rainbow-colored peaks*), Dnase-hypersensitivity areas (*black* and *gray boxes*), genomic location of *PBX1* CpGs corresponding to orange dots in (**a**). Region plot in (**b**) adapted from UCSC genome browser

**Fig. 2:**
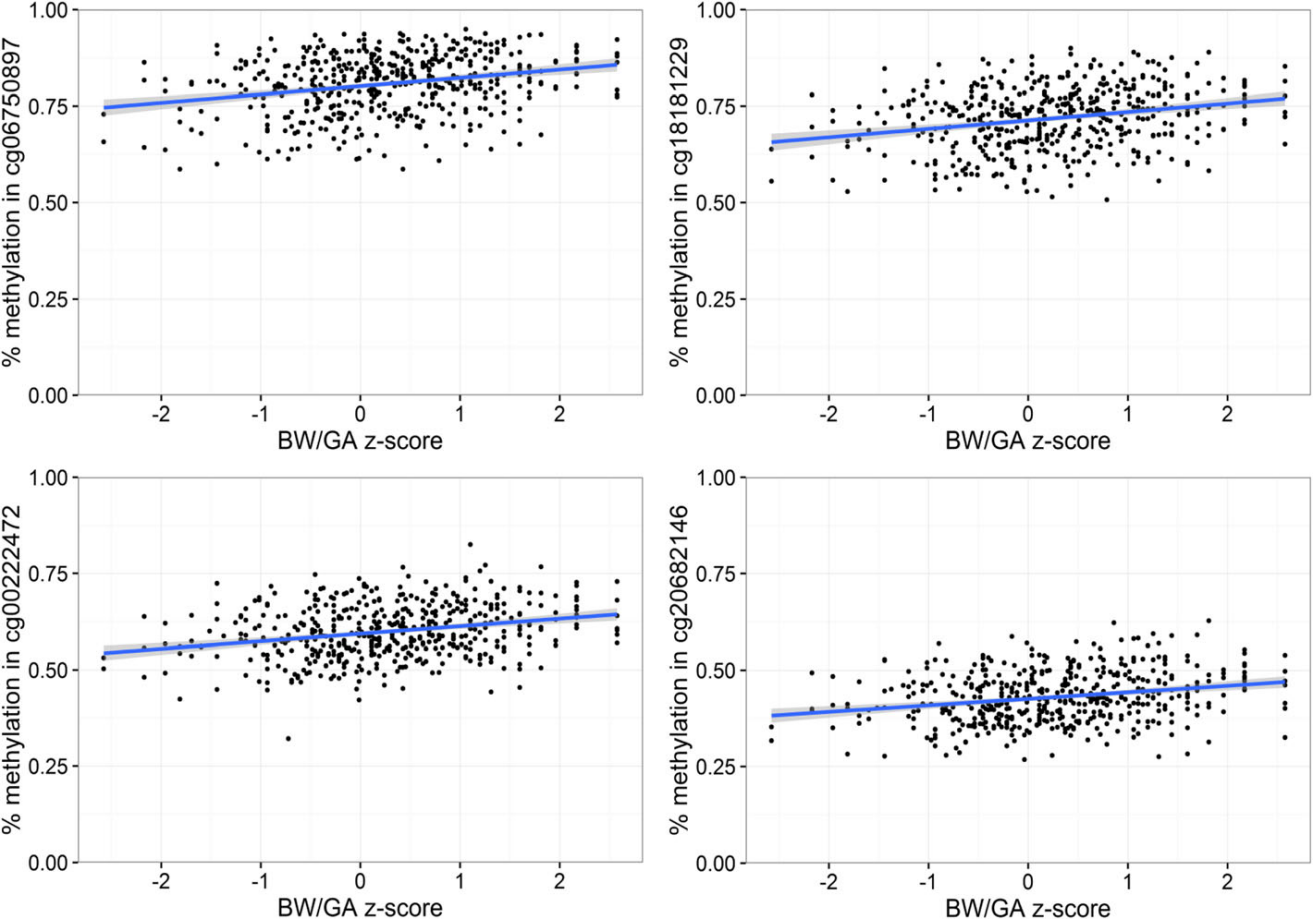
Scatterplots for associations of birth weight-for-gestational-age (BW/GA) with cord blood DNA methylation at 4 CpGs mapped to the *PBX1* gene

In epigenome-wide analyses with multi-variable adjustment, BW/GA was associated (FDR *q* value <0.05) with cord blood DNA methylation at 34 CpG sites (Table 2). Descriptive characteristics of methylation levels at each of these sites are presented in Additional file 1. Of note, higher BW/GA was associated with higher DNA methylation at four CpGs annotated to the pre-B-cell leukemia homeobox 1 (*PBX1*) gene (difference in % methylation (95% CI) for a 1-unit increment in BW/GA *z*-score = 1.9 (1.2, 2.6), 1.9 (1.2, 2.6), 1.8 (1.1, 2.5), and 1.5 (0.9, 2.2) for cg18181229, cg06750897, cg00222472, and cg20682146, respectively; Table 2). At this *PBX1* locus (located on chr 1), cg06750897, cg18181229, cg00222472, and cg20682146 are all located within the same CpG-island region, within the same DNase1 hypersensitivity cluster (ENCODE data, Fig. 1). The scatterplots in Fig. 2 demonstrate linear positive associations between BW/GA and methylation values in these four CpGs, generally with no influence from outlying observations.

**Table 2:** Associations of birth weight-for-gestational age (BW/GA) with DNA methylation sites^a^ in venous umbilical cord blood at delivery, among 476 participants in Project Viva

CpG site^a^	Difference in % methylation for 1-unit increment in BW/GA *z*-score (95% CI)	Nominal *p* value	Gene^b^	Gene region^b^	chr
cg26663636	−0.39 (−0.52, −0.25)	4.31E−09	*NOS1AP*	Body	chr1
cg18181229	1.86 (1.16, 2.56)	1.80E−07	*PBX1*	Body	chr1
cg06750897	1.93 (1.22, 2.64)	1.83E−07	*PBX1*	Body	chr1
cg00222472	1.78 (1.12, 2.45)	2.03E−07	*PBX1*	Body	chr1
cg20682146	1.54 (0.91, 2.17)	1.60E−06	*PBX1*	Body	chr1
cg05780177	0.24 (0.14, 0.35)	2.39E−06	*DENND1B*	TSS200	chr1
cg00325458	0.1 (0.06, 0.14)	7.27E−07	*REL*	TSS200	chr2
cg23483765	0.22 (0.14, 0.31)	1.15E−07	*NIPAL4*	TSS200	chr5
cg24353833	0.42 (0.27, 0.57)	8.38E−08	*NRM*	Body	chr6
cg24641186	0.46 (0.27, 0.64)	1.41E−06	*TFAP2B*	Body	chr6
cg20392842	−1.58 (−2.24, −0.92)	2.54E−06	*HLA-DMB*	TSS200	chr6
cg09364590	0.72 (0.42, 1.03)	3.66E−06	*TIAM2*	Body;TSS200	chr6
cg21809331	0.22 (0.13, 0.31)	2.28E−06	*RBM28*	TSS200	chr7
cg14731462	−0.92 (−1.29, −0.55)	4.20E−07	*PTPRE*	5′UTR	chr10
cg25953130	−2.01 (−2.8, −1.22)	7.76E−07	*ARID5B*	Body	chr10
cg23890469	0.57 (0.34, 0.81)	1.55E−06	*MMRN2*	Body	chr10
cg25124943	−0.92 (−1.31, −0.53)	2.34E−06	*–*	*–*	chr10
cg11606444	0.68 (0.39, 0.98)	4.28E−06	*SORL1*	Body	chr11
cg01345517	0.15 (0.09, 0.21)	1.55E−06	*DERA*	Body	chr12
cg06648759	−1.08 (−1.52, −0.63)	2.02E−06	*–*	*–*	chr13
cg14276580	−0.94 (−1.33, −0.54)	2.24E−06	*–*	*–*	chr13
cg20549688	0.21 (0.13, 0.29)	3.20E−07	*GTF2A2*	TSS200	chr15
cg21842999	0.51 (0.3, 0.72)	3.20E−06	*SHF*	Body	chr15
cg09476997	1.81 (1.06, 2.57)	2.20E−06	*SLC9A3R2*	Body;	chr16
cg27283514	1.51 (0.86, 2.16)	3.34E−06	*–*	*–*	chr16
cg19914554	0.73 (0.47, 0.98)	2.21E−08	*CD7*	1stExon;5′UTR	chr17
cg20186396	0.73 (0.45, 1.02)	3.26E−07	*CD7*	TSS200	chr17
cg14909906	0.17 (0.1, 0.25)	2.37E−06	*KDSR*	1stExon;5′UTR	chr18
cg23882285	0.12 (0.07, 0.17)	3.37E−06	*ROCK1*	TSS200	chr18
cg23026246	0.13 (0.08, 0.19)	2.23E−06	*SPTBN4*	Body	chr19
cg23877608	0.17 (0.1, 0.24)	2.35E−06	*CCDC114*	TSS200	chr19
cg23344780	−0.58 (−0.83, −0.33)	4.10E−06	*EMP3*	5′UTR	chr19
cg04803921	0.13 (0.08, 0.19)	1.68E−06	*HM13*	Body	chr20
cg08422803	1.05 (0.69, 1.41)	8.04E−09	*ITGB2*	TSS200;5′UTR	chr21

*Chr* chromosome, *UTR* untranslated region, *TSS* transcription start site

^a^CpGs are ordered according to chromosome number

^b^Gene and gene region information are according to annotation information from Illumina. Dashed lines indicate that the CpG is annotated to an intergenic region

In addition, we observed that BW/GA was inversely associated (FDR *q* value <0.05) with cord blood DNA methylation at two CpG sites that were also previously reported [22] to show such an association: cg25953130 and cg25124943 (difference in % methylation (95% CI) = −2.0 (−2.8, −1.2) for cg25953130 and −0.9 (−1.3, −0.5) for cg25124943; Table 2).

**Fig. 3:**
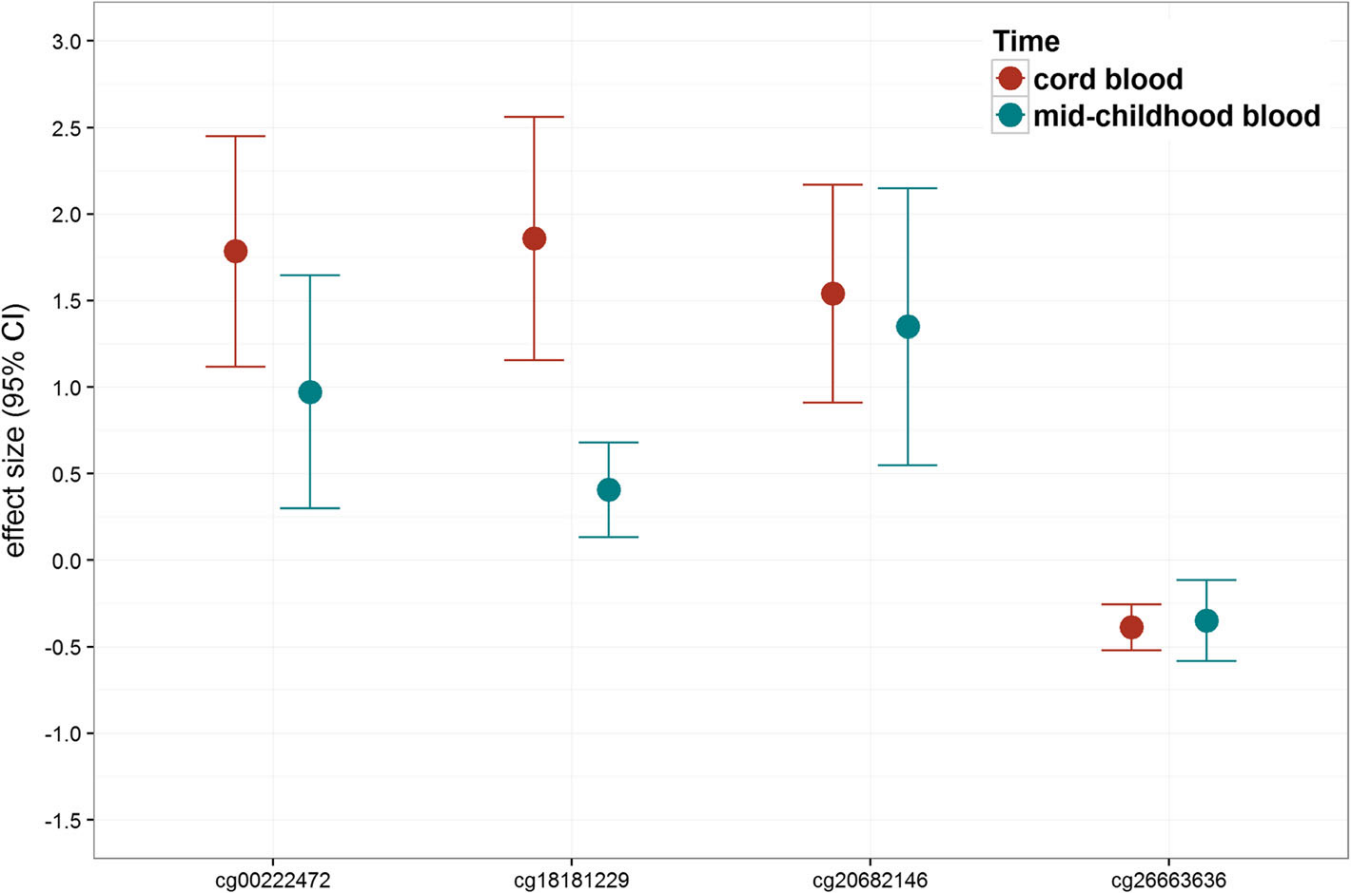
Effect size comparison, for the 4 CpG* sites where birth weight-for-gestational age (BW/GA) was associated with DNA methylation both at birth (cord blood) and mid-childhood (peripheral blood). *CpGs presented map to the following genes: *PBX1* (cg18181229, cg00222472, cg20682146), *NOS1AP* (cg26663636)

**Fig. 4:**
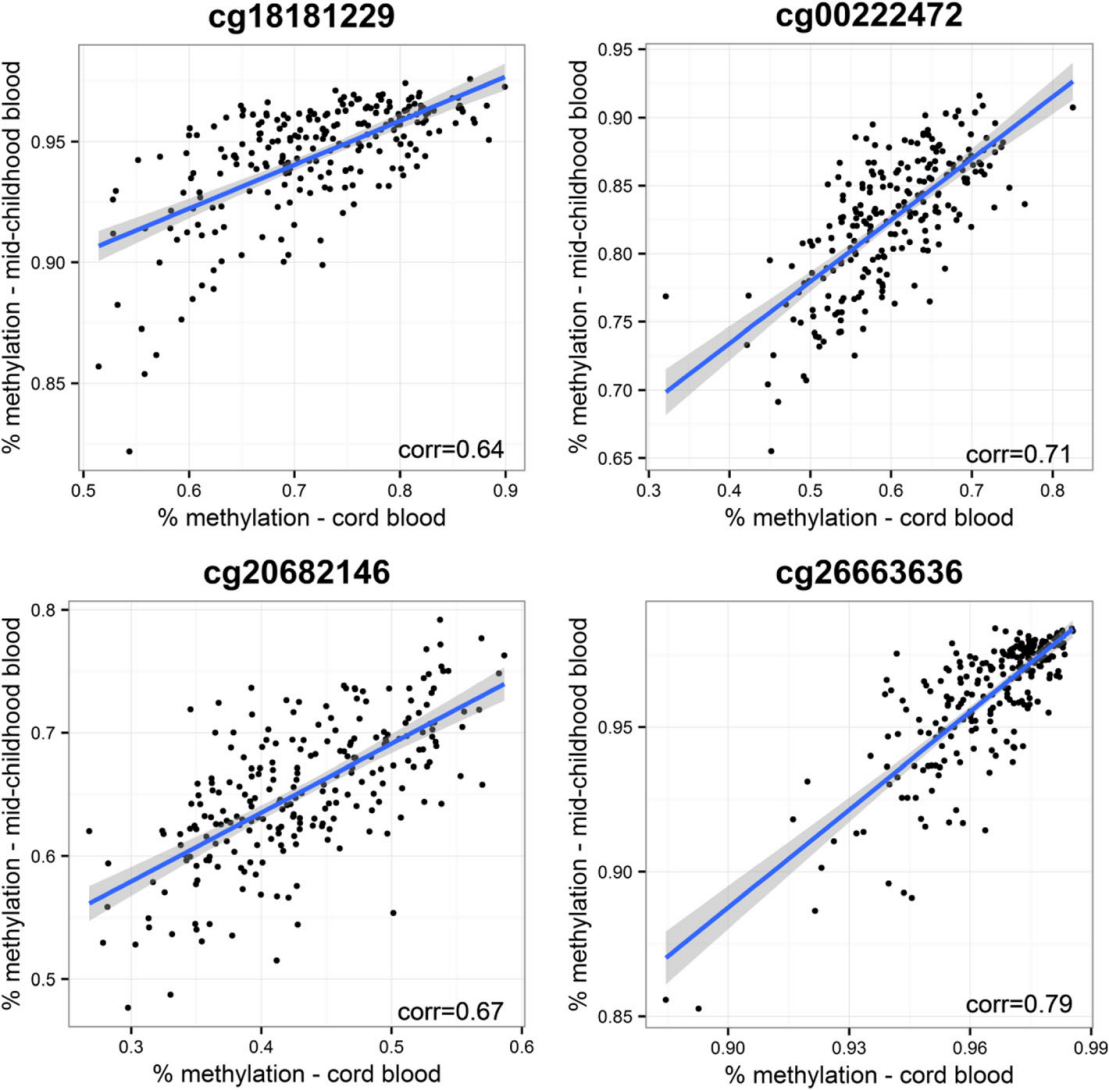
Correlation between cord blood and mid-childhood blood DNA methylation for the four CpG* sites where birth weight-for-gestational age (BW/GA) was associated with DNA methylation both at birth (cord blood) and mid-childhood (peripheral blood). *CpGs presented map to the following genes: *PBX1* (cg18181229, cg00222472, cg20682146), *NOS1AP* (cg26663636)

Finally, we examined whether associations of BW/GA with DNA methylation persisted at mid-childhood. Of the 34 CpG sites where BW/GA was associated with cord blood DNA methylation at birth, associations of BW/GA with blood DNA methylation at mid-childhood remained (FDR *q* value <0.05, for 34 sites tested) for four CpGs: cg26663636, cg18181229, cg00222472, and cg20682146. Notably, cg18181229, cg00222472, and cg20682146 are all annotated to *PBX1*, the locus for which we observed multiple significant CpGs in cord blood analyses, while cg26663636 is annotated to *NOS1AP*. For each of these four CpGs, association of BW/GA with cord blood DNA methylation at birth was consistently in the same direction, and similar in magnitude, as association of BW/GA with peripheral blood DNA methylation at mid-childhood (e.g., difference in % methylation (95% CI) in cg20682146 = 1.5 (0.9, 2.2) at birth and 1.3 (0.5, 2.1) at mid-childhood; Fig. 3). In addition, at each site there was strong correlation between cord blood DNA methylation levels at birth and peripheral blood DNA methylation levels at mid-childhood (Fig. 4).

## Discussion

In this US pre-birth cohort, birth weight-for-gestational age (BW/GA) was associated with cord blood DNA methylation at 34 CpG sites, after adjusting for a range of maternal characteristics and potential biological confounders. Among the 34 sites, we identified four sites where BW/GA was also associated with peripheral blood DNA methylation at mid-childhood, in a manner similar to associations observed at birth.

BW/GA was associated with DNA methylation at birth in four CpG annotated to *PBX1*, and for three of these sites, associations of BW/GA with DNA methylation were also present at mid-childhood. At each of these three sites, the direction and magnitude of the BW/GA-DNA methylation association at birth was similar to that at mid-childhood. Furthermore, the correlation between cord blood DNA methylation at birth and peripheral blood DNA methylation at mid-childhood ranged from 0.64 to 0.71 at these three sites, suggesting that methylation patterns at these sites remain relatively stable with time. *PBX1* encodes a PBX homeobox family transcriptional factor, which acts as part of an important transcriptional network that regulates multiple aspects of embryonic development. *Pbx1*-deficient mice exhibit an embryonic lethal phenotype, characterized by defective development of the spleen, pancreas, kidney, and other organs [39–41]. There is also evidence that pbx1 is required for skeletal patterning and programming [41], and one study found that pbx1 functions within an epigenetic complex that regulates osteoblast differentiation [42]. Specifically, targeted depletion of *PBX1* via short hairpin RNA (shRNA) in bone marrow stromal cells led to increased expression of bone marker genes, increased recruitment of histone acetyltransferases, and decreased H3K9 methylation, reflecting transcriptional activation [42].

Among other CpG sites where BW/GA was associated with cord blood DNA methylation, cg23882285 is annotated to Rho associated coiled-coil containing protein kinase 1(*ROCK1)*, which encodes a protein kinase that is a key regulator of cytoskeleton and cell polarity, and other diverse cellular processes of morphogenesis [43, 44]. Evidence from several studies in mice indicate that ROCK activity is crucial for fetal development, and that mouse spinal neurulation requires precise regulation of ROCK signaling. *ROCK1* knockout mice display an embryonic lethal phenotype, and inactivation of ROCK1 in mice has led to morphological defects and failure of neural tube closure [45, 46].

Two other CpG sites of interest in our cord blood findings were cg25953130 and cg25124943. Associations of birth weight (adjusted for gestational age) and cord blood DNA methylation at these two sites were also previously reported [22] in the MoBa cohort, and similar to their findings, we observed that BW/GA was inversely associated with methylation at these sites. Simpkin et al. [23] (ALSPAC cohort) also reported a similar association for cg25953130. This CpG maps to AT-rich interaction domain 5B (*ARID5B*), which encodes a transcriptional coactivator with a role in adipogenesis. *ARID5B* knockout mice are characterized by reduced lipid accumulation, lower postnatal weight, and a high rate of neonatal death [47].

Aside from the *PBX1* CpG loci described above, cg26663636 was the only site at which BW/GA was associated with both cord blood and mid-childhood peripheral blood DNA methylation. This CpG is annotated to the nitric oxide synthase 1 adaptor protein (*NOS1AP*) locus, which encodes an adapter and regulator of the neuronal nitric oxide synthase (nNOS) enzyme implicated in modulating physiological functions such as learning, memory, and neurogenesis [48]. In addition to constituting the major source of NO in neurons, nNOS is present in skeletal muscle, cardiac muscle, and smooth muscles, where NO controls blood flow and muscle contractility. In particular, nNOS is an important cardiac protector in the heart, ensuring regulation of functions when the heart is under stress [48, 49].

In the ALSPAC cohort, Simpkin et al. reported that birth weight (adjusted for gestational age) was associated with cord blood methylation in 23 CpG sites. For these specific CpGs, they further used serially measured DNA methylation at birth, ages 7 and 17 to longitudinally model methylation changes over time. They observed that the majority of these CpGs showed marked changes in methylation levels during childhood, and that lower birth weight was associated with faster changes in methylation levels, suggesting that there is erasure of birth weight-related cord blood DNA methylation signatures with time [23]. Given that our analyses involved DNA methylation at two time points, we did not use longitudinal modeling to examine BW/GA-associated changes in methylation over time. Rather, we asked whether there were CpG sites where BW/GA was associated with DNA methylation both at birth and mid-childhood. We observed this to be the case, but only for four of the 34 sites that we tested. Thus, our results do not definitively differ from Simpkin et al.’s; rather, they suggest that persistence of associations may be dependent on specific sites examined.

Our study has several strengths. We conducted a comprehensive epigenome-wide investigation of BW/GA, the first in a relatively large sample from a US pregnancy prospective cohort. We used a nearly continuous measure of birth weight adjusted for gestational age (created using Nationwide US Natality datasets), which has the advantage of not assuming a linear relationship between birth weight and gestational age [30]. Furthermore, we examined DNA methylation at two time points. In addition, we used a cord blood reference panel to estimate cell type proportions in cord blood, reducing the possibility of reporting spurious DNA methylation associations due to varying cell type proportions. Given that contamination by nRBCs is still possible in isolated buffy coat, using a cord blood reference panel that accounts for presence of nRBCs further helps to reduce residual confounding; this is an advantage over prior cord blood epigenetic studies that have used the adult whole blood reference panel to estimate and account for cell type proportions. Although our DNA methylation analyses were on buffy coat isolated from whole cord blood, it would be interesting for future epigenetic studies to investigate methylation profiles in isolated nRBCs. Increased concentrations of nRBCs at birth have been observed in relation to maternal chronic conditions [37, 50–53] and can be predictive of child health and future neurodevelopment [54]. Thus, examining the methylation patterns in nRBCs may provide further insight on fetal development and later health and disease. However, isolation of DNA from nRBCs can prove challenging [27]. A limitation of our study is that the relatively higher socioeconomic status in Project Viva may reduce the generalizability of our findings. Furthermore, the Illumina Infinium 450K array has until recently been the most popular and feasible choice for epigenome-wide analyses; however, it approximately covers only 1.5% of genomic CpGs and is heavily geared towards coverage of gene promoter regions and protein-encoding genes [55]. The recently released 850K EPIC array covers an additional 413,745 new CpG sites which are enriched in regulatory regions such and “open” chromatin regions [56]. Recent evidence highlights the important role of DNA methylation in such regulatory and non-coding genomic regions [57, 58] and its relevance to disease [59].

## Conclusions

In conclusion, we observed that birth weight-for-gestational age was associated with DNA methylation patterns at birth at select CpG sites; for several of these sites, birth weight-for-gestational age was also associated with DNA methylation at mid-childhood. We were also successful in replicating some findings from prior studies in European cohorts. Given that cardio-metabolic abnormalities associated with fetal growth often do not manifest early in life, identifying the underlying molecular markers associated with fetal growth may help to better elucidate the early development of long-term risk. Further research will better clarify the extent to which DNA methylation signatures of fetal growth and development persist with time beyond childhood, and the extent to which they are related to cardio-metabolic dysregulation.

## Additional file

Additional file 1: Descriptive characteristics of methylation levels in birth weight-for-gestational age (BW/GA)-associated DNA methylation sites in venous umbilical cord blood at delivery, among 476 participants in Project Viva. (XLSX 12 kb)

## Abbreviations

BMIQ: Beta-Mixture Quantile dilation
BW/GA: Birth weight-for-gestational age
FDR: False discovery rate
LGA: Large-for-gestational age
nRBCs: Nucleated red blood cells
SGA: Small-for-gestational age
